# Establishment of age- and -gender specific reference intervals for amino acids and acylcarnitines by tandem mass spectrometry in Turkish paediatric population

**DOI:** 10.11613/BM.2023.030704

**Published:** 2023-10-15

**Authors:** Özlem Çakır Madenci, Soner Erdin, Ayşe Kestane, Müge Kutnu

**Affiliations:** Department of Biochemistry, Kartal Dr. Lütfi Kırdar City Hospital, Istanbul, Turkey

**Keywords:** inborn errors of metabolism, laboratory information system (LIS), paediatrics, reference intervals, tandem mass spectrometry

## Abstract

**Introduction:**

We determined age- and gender-specific reference intervals (RIs) for acylcarnitines and amino acids by tandem mass spectrometry (MS/MS) in the Turkish paediatric population by using laboratory information system (LIS) data.

**Materials and methods:**

A total of 9156 MS/MS results of children between 0-18 years of age, were downloaded from the LIS. Premature infants and newborns followed in the intensive care unit were excluded and only the first result of each patient attending outpatient clinics was included. Children with a known or suspected diagnosis of metabolic disease, malignancy, epilepsy, mental retardation, or genetic disorder were excluded. Laboratory results were evaluated and children with any pathological laboratory finding were excluded, resulting in a final sample size of 3357 (2029 boys and 1328 girls). Blood was collected by capillary puncture and spotted on Whatman 903 filter paper cards and analysed by MS/MS (Shimadzu LCMS-8050, Shimadzu Corporation, Kyoto, Japan). Data were evaluated for age and gender differences and age partitioning was performed according to the literature and visual evaluation of the data. Age subgroups were: ≤ 1 month, 2 months-1 year, 2-5 years, 6-10 years, and 11-18 years.

**Results:**

There were significant age-related differences for the majority of amino acids and acylcarnitines thus age dependent RIs were established. Gender-specific RIs were established for tyrosine, leucine-isoleucine, isovalerylcarnitine (C5) and hexadecanoylcarnitine (C16).

**Conclusions:**

Establishing age-related RIs can enhance the quality of medical care by facilitating early diagnosis and therapy, especially in certain metabolic disorders presenting with mild biochemical abnormalities and subtle clinical manifestations.

## Introduction

Inborn errors of metabolism (IEM) are serious, degenerative, chronic diseases that can cause mental retardation and various degrees of physical disability, and the fundamental issue for these individuals is that they are frequently misdiagnosed or diagnosed late owing to the lack of specialized laboratories (*1*). Early diagnosis and prompt initiation of therapy can enhance the quality of life for those who are affected (*2*, *3*).

Advanced newborn screening (NBS) by tandem mass spectrometry (MS/MS) is performed to identify amino acidopathies, fatty acid oxidation disorders, and organic acid disorders (*4*). A positive screening result may not always indicate that the infant has a metabolic disorder. Positive test results should be repeated from the original sample and when the repeated result is again positive then follow-up diagnostic tests, such as plasma amino acids, acylcarnitine analysis and urine organic acid analysis, should be performed to confirm the screening result and avoid false positives (*5*). Using the MS/MS technique, it is possible to simultaneously detect and identify multiple analytes from a single dried blood spot with high sensitivity, accuracy, and precision (*1*). Individual IEMs are rare disorders, most having an incidence of less than 1 *per* 100,000 births. However, when considered collectively, the incidence may approach 1 in 800 to 1 in 2500 births (*6*). In Turkey, the prevalence of IEM would be expected to be higher partly due to consanguineous marriages. However, extended NBS is not included in the national health program, and only clinically suspected individuals of any age are subjected to screening with dried blood spots for amino acid and acylcarnitine profile (*7*).

Reference intervals (RIs), which represent the central 95% of the data from the reference population, are critical for accurate interpretation of laboratory results. It is recommended that laboratories establish their RIs in their local population using either the direct or indirect method, along with their specific techniques and instruments (*8*, *9*). Although the Clinical and Laboratory Standards Institute (CLSI) recommends the direct approach, it can be challenging to use in routine practice due to difficulties in accessing healthy controls, especially in the paediatric population (*9*). The laboratory information system (LIS) is used to retrieve laboratory data for the indirect method. Although it is more practical and cost-effective, cautious selection should be done to avoid including results from unhealthy individuals in database (*9*, *10*). Since the analytical and biological variability of parameter is also taken into consideration, the results of the indirect method are much closer to the population’s actual status in a particular area (*8*). The International Federation of Clinical Chemistry and Laboratory Medicine’s (IFCC) committee on RIs and decision limits has approved the use of indirect methods to establish and verify RIs (*9*).

Cut-offs of disease-specific biomarkers are used for diagnosis of particular metabolic diseases (*11*). The lower and upper cut-offs for the primary markers have been set below the 1st percentile and above the 99th percentile, respectively. The Recommended Uniform Screening Panel (RUSP) has validated the cut-offs for the primary and secondary markers for screening amino acidopathies, fatty acid oxidation disorders, and organic acid disorders (*12*). Once age-specific cut-offs have been established, laboratories should carefully monitor whether age-specific cut-offs are compatible with the patient’s clinic by evaluating false positive rates (*13*). In Turkish population, age-dependent RI for amino acids and acylcarnitines from dried blood spots were established in two studies that included LIS data for children under and over 1 year of age and newborns (*14*, *15*).

In the present study, we aimed to determine age- and gender-specific RIs for acylcarnitines and amino acids by tandem MS/MS in Turkish paediatric population by analysing LIS data from a hospital laboratory serving a large population. Establishing age-specific RIs for amino acids and acylcarnitines may help to improve the quality of healthcare for affected individuals through early diagnosis and treatment.

## Material and methods

### Study design

Age- and gender-specific RIs for 10 amino acids (alanine, glycine, leucine-isoleucine, methionine, ornithine, phenylalanine, tyrosine, valine, arginine, and citrulline) and 22 acylcarnitines: free carnitine (C0), acetylcarnitine (C2), propionylcarnitine (C3), malonylcarnitine (C3DC), butrylcarnitine (C4), isovalerylcarnitine (C5), tiglycarnitine (C5:1), glutarylcarnitine (C5DC), 3-hydroxyisovalerylcarnitine (C5-OH), hexanoylcarnitine (C6), octanoylcarnitine (C8), decanoylcarnitine (C10), decenoylcarnitine (C10:1), dodecanoylcarnitine (C12), tetradecanoylcarnitine (C14), tetradecenoylcarnitine (C14:1), hexadecanoylcarnitine (C16), hexadecenoylcarnitine (C16:1), 3-OH-hexadecanoylcarnitine (C16-OH), stearoylcarnitine (C18), oleylcarnitine (C18:1) and 3-OH-octadecanoylcarnitine (C18-OH) were determined by an indirect method in dried blood spots by MS/MS. The study was conducted according to the CLSI C28-A3 protocol (*9*) at Dr. Lütfi Kırdar City Hospital, which provides health care with 1205 inpatient beds and 10,000 daily outpatient visits. Our central laboratory analyses about 15,000 samples daily from 8 other hospitals and 166 primary care centers, serving a large population living in both regions of Istanbul.

### Subjects

A total of 9156 MS/MS results from children between 0 and 18 years of age, who were admitted to our laboratory between January 2021 and January 2023, were downloaded from the LIS. The study included patients attending outpatient clinics. Information on patients’ general health status was obtained from the hospital information system records. However, premature infants, new-borns followed in the intensive care unit, and children with a known or suspected diagnosis of metabolic disease, malignancy, epilepsy, mental retardation, or genetic disorder were excluded. Furthermore, children with any pathological laboratory findings (*e.g.*, urine ketone, routine biochemistry findings, ammonia, blood gases, urine organic acids, plasma amino acids, biotinidase activity, or homocysteine) were also excluded.

**Figure 1:**
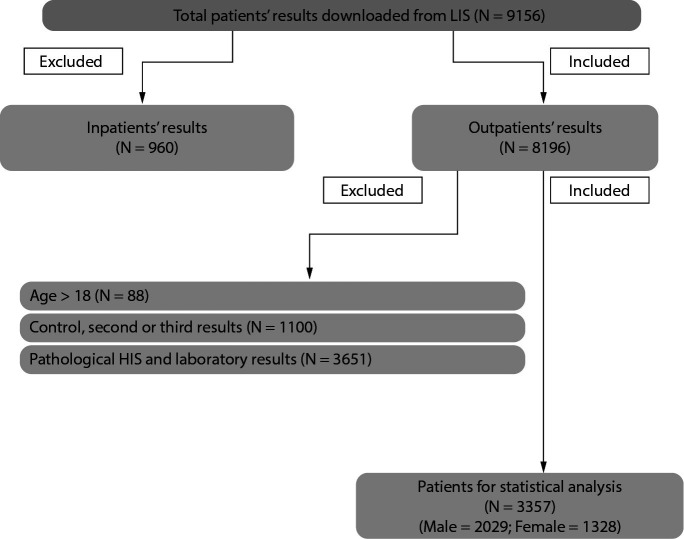
The flow diagram of the study.

Amino acid and acylcarnitine concentrations were evaluated, and each analyte concentration was plotted against age for both genders. Outliers were removed, resulting in a final sample size of 3357 (2029 males and 1328 females) for statistical analyses. The data were inspected for age and gender differences. Age partitioning was performed according to the visual evaluation of the data and in line with the current literature (*2*, *14*, *15*). The age groups were as follows: group A: ≤ 1 month, group B: 2 months-1 year, group C: 2-5 years, group D: 6-10 years, and group E: 11-18 years. The flow diagram of the study is shown in Figure 1. This study was approved by our institution’s Ethical committee on 12th April 2023 (Approval number: 2023/514/247/5).

### Blood sampling

Blood was collected by capillary puncture and spotted on filter paper cards (Whatman 903 filters, GE Healthcare, Westborough, USA) which were dried at room temperature for at least 4 hours, according to the CLSI standard (*16*).

### Methods

Dried blood spots (DBS) were punched out to a diameter of 3.2 mm and placed in a single well of a 96-well polystyrene plate, to which 100 μL of the daily working solution containing internal standards for amino acids and acylcarnitines was added. The plate was sealed using adhesive sealing film and shaken at 45 °C for 45 minutes. The extracts were transferred to a new polystyrene 96 - microwell plate and then dried at 50 °C under a stream of nitrogen. Then 50 μL of methanol was added and evaporated again under a stream of nitrogen. The samples were then reconstituted in 50 μL n-butanol 3N HCl and incubated at 65 ˚C for 20 min. The resulting mixtures were again evaporated for approximately 20 min and each residue was finally reconstituted in 100 μL of mobile phase, covered with aluminium foil and analysed by MS/MS (Shimadzu LCMS-8050, Shimadzu Corporation, Kyoto, Japan) analyser. The derivatized MS/MS commercial kit (Obikron, Ankara, Turkey) was used. Internal standards were obtained from Cambridge isotope laboratories (Tewksbury, USA). Quantification of target analytes was calculated by comparing the ion abundance ratios of each pure analyte with the ratios of the isotopically labelled internal standards. For internal quality control, four levels of quality control materials for amino acids and acylcarnitines were obtained from the Centers for Disease Control and Prevention (CDC, Atlanta, USA) and included in every batch. The interassay precision and coefficient of variation (CV%) were found to be < 10% for both amino acids and acylcarnitines. Our laboratory actively participates in the Erndim external quality assurance programme, which supplies 2 sets and 6 different proficiency DBS samples each year. The performance of laboratories is monitored using both quantitative and qualitative schemes, which focus on numerical data and clinical interpretation, respectively.

### Statistical analysis

The non-parametric rank-based method recommended by the IFCC and CLSI was used to estimate the RIs by using the percentile distribution of the metabolites. Each analyte concentration was plotted against age for both genders. Data distribution was visually inspected and Shapiro-Wilk test was used to assess normality. Logarithmic transformations were performed to improve normality and then outliers were subsequently eliminated by Tukey’s method until none remained. Gender differences were assessed using the standard normal deviation test (z-test) and the z-value was calculated and compared to a “critical” z-value. For those parameters whose z-value exceeds the critical z-value, a gender-dependent RI is established. For age partitioning, subgroups were compared using ANOVA and *post hoc* tests. If no significant difference was observed age subgroups were combined where appropriate. Many metabolites differed by age and age-specific RIs were determined for them. Age and gender-specific 1st, 2.5th, 97.5th, and 99th percentile values were reported, P < 0.05 was considered statistically significant. MedCalc version 19.2.1 (MedCalc Software Ltd, Ostend, Belgium) software was used for the statistical calculations.

## Results

Gender-specific reference ranges were established for tyrosine and leucine-isoleucine. Girls in the ≤ 1 month age group had higher tyrosine concentrations, while leucine-isoleucine concentrations were higher in boys in the 11-18 years age group. For acylcarnitines, gender-specific reference ranges were established for C5 and C16 in the ≤ 1 month and 11-18 years age groups respectively with boys having higher values for both parameters. Regarding amino acids, more pronounced age-specific variations were observed, and the majority (glycine, leucine-isoleucine, ornithine, and tyrosine) showed higher concentrations in the ≤ 1 month age group. On the other hand, the 99th percentile cut-off value for arginine was higher in the age range of 11 to 18 years compared to infants. As for acylcarnitines, concentrations of C0, C5-OH, C10, and C10:1 were lower in infants and increased with age, while concentrations of C2, C3, C5, C6, C12, C14, C16, C16:1, C18, and C18:1 decreased with age. The age-gender-specific 1st to 99th and 2.5th to 97.5th percentile distributions of amino acids are presented in Table 1, while age-gender-specific 1st to 99th and 2.5th to 97.5th percentile distributions of acylcarnitines are presented in Table 2.

**Table 1:** Age-gender specific distribution of aminoacids

**Aminoacid**	**Age group**	**Percentiles (µmol/L)**			
		**1st (90% CI)**	**99th (90% CI)**	**2.5th (90% CI)**	**97.5th (90% CI)**
Alanine	All group (N = 3336)	120 (110-128)	539 (518-549)	148 (146-149)	451 (442-462)
	≤ 1 month (N = 452)	129 (123-134)	516 (493-540)	151 (143-158)	437 (425-456)
	> 1 month - ≤ 1 year (N = 1021)	111 (100-126)	530 (504-583)	146 (138-149)	431 (411-452)
	1-5 years (N = 1012)	119 (99-128)	544 (489-560)	146 (141-149)	445 (433-464)
	5-10 years (N = 518)	127 (122-132)	553 (532-576)	150 (136-161)	460 (438-495)
	10-18 years (N = 333)	152 (145-161)	602 (569-635)	175 (163-19)	521 (480-545)
Glycine	All group (N = 3351)	130 (126-138)	602 (571-656)	157 (153-159)	478 (458-494)
	≤ 1 month (N = 453)	147 (141-154)	633 (602-665)	181 (171-193)	586 (548-654)
	> 1 month - ≤ 1 year (N = 1027)	119 (107-130)	597 (533-668)	142 (138-148)	454 (425-479)
	1-5 years (N = 1016)	129 (116-142)	538 (483-729)	159 (157-165)	440 (415- 453)
	5-10 years (N = 520)	144 (139-150)	480 (462-499)	171 (158-178)	453 (398-506)
	10-18 years (N = 335)	162 (155-170)	515 (491-540)	198 (184-210)	482 (449-535)
Leucine- Isoleucine*	All group (N = 3353)	57.4 (54.8-59.7)	243 (234-249)	65.9 (64.6-67.9)	207.2 (203.6-212.4)
	≤ 1 month (N = 454)	80.4 (76.7-84.5)	301 (288-315)	82.4 (77.2-88.7)	241.6 (231.8-249.1)
	> 1 month - ≤ 1 year (N = 1028)	54.8 (48.4-59.3)	231 (223-257)	65.2 (62.0-71.0)	203.9 (196.8-211.3)
	1-10 years (N = 1536)	57.3 (52.0-59.6)	224 (216-248)	64.0 (62.1-66.8)	186.5 (180.7-189.9)
	10-18 years boy (N = 196)	60.6 (57.2-64.2)	227 (214-241)	72.0 (65.6-79.1)	200.5 (182.3-207.6)
	10-18 years girl (N = 139)	56.5 (52.8-61.4)	189 (174-204)	66.0 (58.2-72.8)	182.8 (161.8-224.4)
Methionine	All group (N = 3349)	10.7 (10.3-11.3)	50.4 (47.2-53.8)	12.5 (12.3-12.7)	39.4 (38.6-40.9)
	≤ 1 month (N = 454)	12.1 (11.6-12.7)	47.2 (45.1-49.3)	13.5 (12.6-14.9)	41.0 (38.5-44.6)
	> 1 month - ≤ 1 year (N = 1026)	10.2 (10.0-11.1)	53.0 (47.1-63.6)	12.4 (12.0-12.7)	40.0 (38.1-43.3)
	1-10 years (N = 1534)	10.6 (10.1-11.1)	51.2 (47.0-59.0)	12.2 (11.9-12.5)	39.2 (37.1-41.2)
	10-18 years (N = 335)	11.4 (10.9-12.0)	40.0 (38.0-42.1)	13.6 (12.9-14.7)	37.1 (34.7-40.8)
Ornithine	All group (N = 2771)	32. 9 (30.7-39,6)	24 (218-249)	46.2 (44.7- 48.1)	182.6 (178.5-190.0)
	≤ 1 year (N = 1232)	33.2 (22.8-37.7)	243 (228-269)	46.3 (43.9- 50.4)	197.5 (188.14-207.9)
	1-5 years (N = 849)	30.7 (28.0-34.1)	242 (208-313)	44.0 (39.3-46.7)	172.9 (159.9-180.7)
	5-18 years (N = 4690)	39.2 (31.0-44.8)	208 (184-249)	50.1 (46.6-52.4)	171.1 (162.7-181.7)
Phenylalanine	All group (N = 3354)	27.2 (24.9-28.9)	96.4 93.8-107)	31.8 (30.8-31.8)	81.7 (79.2-84.2)
	≤ 1 month (N = 454)	28.7 (27.6-29.9)	87.6 (84.2-91.0)	33.0 (31.2-34.4)	74.4 (72.7-83.3)
	> 1 month - ≤ 1 year (N = 1028)	24.5 (21.2-25.6)	105 (90.6-128)	29.9 (28.0-30.0)	81.2 (76.8-84.6)
	1-18 years (N = 1872)	30.0 (30.0-31.0)	95.1 (93.0-106)	32.9 (32.3-33.7)	84.0 (79.9-85.9)
Tyrosine*	All group (N = 3352)	29.4 (27.3-30.0)	197 (191-208)	32.1 (31.2-33.3)	151.1 (143.7-156.6)
	≤ 1 month boy (N = 254)	35.8 (32.9-39.0)	238 (219-257)	40.2 (31.4-45.5)	176.6 (167.1-193.8)
	≤ 1 month girl (N = 200)	37.1 (33.4-41.3)	285 (256-316)	43.4 (31.4-53.5)	200.2 (192.2-226.2)
	>1 month - ≤ 1 year (N = 1026)	28.1 (25.8-30.0)	198 (188-270)	32.8 (30.7-34.9)	148.4 (134.8-155.0)
	1-18 years (N = 1872)	29.4 (26.8-30.0)	169 (142-204)	31.1 (30.0-32.2)	113.0 (109.3-118.0)
Valine	All group (N = 3351)	66.9 (63.5-69.1)	275 (268-285)	76.8 (75.5-79.0)	226. (220.8-233.9)
	≤ 1 year (N = 1480)	61.4 (56.4-65.6)	258 (237-282)	70.8 (69.4-73.1)	203.2 (196.4- 211.6)
	1-18 years (N = 1871)	81.6 (75.2-85.8)	284 (269-290)	93.9 (92.4-95.7)	238. (233.3- 246.1)
Arginine	All group (N = 3333)	3.90 (3.71-4.17)	60.4 (57.6-62.9)	5.00 (5.00-5.00)	49.04 (47.05-50.47)
	≤ 1 month (N = 452)	2.47 (2.28-2.69)	36.2 (32.9-39.7)	4.50 (4.00-4.98)	29.49 (26.16-32.94)
	>1 month - ≤1 year (N = 1023)	3.89 (3.58-4.32)	59.6 (56.2-60.9)	5.00 (5.00-5.02)	47.32 (44.90-50.44)
	1-18 years (N = 1858)	4.21 (2.77-4.63)	61.9 (59.0-63.7)	5.00 (5.00-5.27)	50.75 (49.42-52.64)
Citrulline	All group (N = 3350)	7.44 (7.14-7.70)	42.1 (41.1-45.6)	9.21 (8.96-9.48)	37.25 (36.38-37.84)
	≤1 month (N = 453)	6.48 (6.17-6.83)	31.8 (30.2-33.5)	8.30 (7.46-8.67)	28.19 (25.75-29.57)
	>1 month - ≤ 1 year (N = 1028)	7.13 (6.21-7.39)	42.0 (37.7-48.8)	8.13 (7.83-8.64)	31.87 (30.81-33.65)
	1-5 years (N = 1015)	9.64 (7.59-10.5)	45.5 (41.3-58.8)	12.34 (11.49-13.34)	37.61 (36.52-38.97)
	5-18 years (N = 854)	11.8 (9.17-12.8)	43.6 (40.5-50.4)	14.11 (13.42-14.90)	39.06 (38.65-39.78)
*Difference across gender groups. CI - confidence interval.					

**Table 2:** Age-gender distribution of acylcarnitines

**Acylcarnitine**	**Age groups**	**Percentiles (µmol/L)**			
		**1st (90% CI)**	**99th (90% CI)**	**2.5th (90% CI)**	**97.5th (90% CI)**
C0	All group (N = 3350)	14.0 (13.0-14.5)	70.6 (68.5-73.0)	17.62 (17.07-17.97)	61.26 (59.90-63.01)
	≤ 1 month (N = 453)	11.9 (11.3-12.6)	59.5 (56.5-62.6)	14.10 (12.96-14.78)	47.69 (45.03-54.09)
	> 1month - ≤ 1 year (N = 1026)	14.6 (13.4-16.6)	73.2 (71.2-76.0)	19.28 (17.74-20.89)	66.25 (64.60-68.57)
	1-18 years (N = 1871)	15.1 (14.6-16.2)	67.2 (63.9-69.6)	18.29 (17.84-19.02)	55.18 (53.44-57.57)
C2	All group (N = 3351)	7.52 (7.50-7.74)	43.6 (41.2-47.8)	8.37 (8.15-8.48)	33.76 (32.65-34.72)
	≤ 1 month (N = 454)	5.09 (4.80-5.41)	41.9 (39.0-45.1)	7.91 (7.61-8.07)	36.78 (33.64-41.24)
	> 1 month - ≤ 1 year (N = 1027)	7.80 (7.51-7.99)	49.2 (44.4-59.8)	8.78 (8.43-9.29)	38.50 (36.52-40.92)
	1-5 years (N = 1015)	7.50 (7.50-7.77)	36.5 (32.4-40.8)	8.57 (8.17-8.88)	29.55 (28.53-30.60)
	5-18 years (N = 855)	7.56 (7.50-7.77)	34.2 (30.1-45.3)	8.17 (7.87-8.48)	26.66 (25.20-28.24)
C3	All group (N = 3343)	0.47 (0.43-0.52)	3.61 (3.47-3.75)	0.66 (0.64-0.68)	3.22 (3.15-3.26)
	≤ 1 month (N = 454)	0.37 (0.34-0.39)	4.73 (4.38-5.09)	0.54 (0.47-0.57)	3.28 (3.13-3.42)
	> 1 month - 18 years (N = 2889)	0.50 (0.43-0.57)	3.62 (3.47-3.73)	0.71 (0.69-0.74)	3.19 (3.15-3.25)
C3DC	All group (N = 1924)	0.02 (0.02-0.02)	0.25 (0.25-0.25)	0.04 (0.03-0.04)	0.22 (0.21-0.23)
	≤ 1 month (N = 245)	0.02 (0.02-0.03)	0.30 (0.25-0.33)	0.04 (0.02-0.04)	0.19 (0.18-25)
	>1 month - 5 years (N = 1196)	0.02 (0.02-0.02)	0.25 (0.25-0.25)	0.04 (0.03-0.04)	0.23 (0.22-0.24)
	5-10 years (N = 305)	0.03 (0.03-0.04)	0.38 (0.32-0.41)	0.04 (0.03-0.04)	0.22 (0.20-0.25)
	10-18 years (N = 178)	0.00 (0.00-0.00)	0.23 (0.22-0.24)	0.03 (0.00-0.05)	0.22 (0.20-0.24)
C4	All group (N = 3353)	0.08 (0.08-0.08)	0.80 (0.77-0.86)	0.10 (0.10-0.10)	0.59 (0.57-0.62)
	0-5 years (N = 2498)	0.08 (0.08-0.09)	0.81 (0.78-0.87)	0.10 (0.10-0.10)	0.59 (0.57-0.63)
	5-18 years (N = 855)	0.08 (0.05-0.08)	0.77 (0.71-1.14)	0.10 (0.09-0.10)	0.58 (0.53-0.65)
C5*	All group (N = 3354)	0.06 (0.05-0.06)	0.34 (0.31-0.36)	0.07 (0.07-0.07)	0.26 (0.25-0.27)
	≤ 1month boy (N = 254)	0.06 (0.05-0.07)	0.34 (0.32-0.38)	0.09 (0.08-0.09)	0.33 (0.29-0.38)
	≤ 1month girl (N = 200)	0.07 (0.06-0.08)	0.35 (0.33-0.39)	0.09 (0.08-0.10)	0.30 (0.27-0.36)
	>1 month - ≤ 1 year (N = 1028)	0.06 (0.05-0.06)	0.33 (0.30-0.36)	0.07 (0.070-0.07)	0.25 (0.24-0.27)
	1-5 years (N = 1016)	0.05 (0.05-0.06)	0.29 (0.27-0.32)	0.07 (0.06-0.07)	0.24 (0.23-0.26)
	5-10 years (N = 521)	0.04 (0.04-0.05)	0.25 (0.23-0.27)	0.07 (0.06-0.07)	0.23 (0.21-0.25)
	10-18 years (N = 335)	0.05 (0.04-0.06)	0.27 (0.26-0.30)	0.07 (0.06-0.07)	0.26 (0.23-0.32)
C5:1	All group (N = 3279)	0.00 (0.00-0.01)	0.13 (0.12-0.17)	0.01 (0.01-0.01)	0.08 (0.08-0.09)
	≤ 12 months (N = 1449)	0.00 (0.00-0.01)	0.12 (0.10-0.17)	0.01 (0.01-0.01)	0.08 (0.08-0.09)
	1-18 years (N = 1830)	0.00 (0.00-0.01)	0.13 (0.12-0.18)	0.01 (0.01-0.01)	0.09 (0.08-0.10)
C5DC	All Group (N = 3354)	0.03 (0.03-0.03)	0.26 (0.24-0.28)	0.04 (0.04-0.04)	0.20 (0.19-0.20)
	0-5 years (N = 2498)	0.03 (0.03-0.03)	0.25 (0.24-0.28)	0.04 (0.04-0.04)	0.19 (0.19-0.20)
	5-18 years (N = 856)	0.03 (0.02-0.04)	0.28 (0.25-0.33)	0.04 (0.04-0.05)	0.21 (0.19-0.22)
C5-OH	All group (N = 3356)	0.06 (0.06-0.07)	0.53 (0.50-0.59)	0.08 (0.08-0.08)	0.39 (0.38-0.41)
	≤ 1 month (N = 454)	0.06 (0.05-0.06)	0.31 (0.28-0.32)	0.07 (0.07-0.08)	0.25 (0.24-0.27)
	> 1month - ≤ 1 year (N = 1030)	0.06 (0.05-0.06)	0.39 (0.36-0.45)	0.07 (0.07-0.08)	0.30 (0.28-0.33)
	1-10 years (N = 1537)	0.07 (0.06-0.08)	0.56 (0.52-0.70)	0.09 (0.09-0.10)	0.41 (0.39-0.43)
	10-18 years (N = 335)	0.08 (0.07-0.09)	0.63 (0.58-0.69)	0.10 (0.08-0.11)	0.51 (0.47-0.58)
C6	All group (N = 3325)	0.02 (0.02-0.02)	0.28 (0.26-0.30)	0.03 (0.03-0.03)	0.21 (0.20-0.22)
	≤ 12 months (N = 1468)	0.02 (0.02-0.03)	0.29 (0.26-0.32)	0.03 (0.03-0.03)	0.21 (0.19-0.22)
	1-18 years (N = 1857)	0.02 (0.02-0.02)	0.27 (0.26-0.31)	0.03 (0.03-0.03)	0.22 (0.19-0.24)
C8	All group (N = 3343)	0.02 (0.02-0.02)	0.24 (0.21-0.24)	0.03 (0.02-0.03)	0.16 (0.15-0.17)
	≤ 1 month (N = 454)	0.01 (0.01-0.02)	0.13 (0.12-0.14)	0.03 (0.02-0.03)	0.11 (0.10-0.15)
	> 1 month - ≤ 1 year (N = 1026)	0.02 (0.02-0.02)	0.19 (0.17-0.24)	0.03 (0.02-0.03)	0.14 (0.13-0.16)
	1-5 years (N = 1009)	0.02 (0.02-0.02)	0.26 (0.19-0.28)	0.03 (0.02-0.03)	0.16 (0.14-0.18)
	5-18 years (N = 854)	0.03 (0.02-0.02)	0.24 (0.24-0.25)	0.03 (0.02-0.03)	0.19 (0.17-0.21)
C10	All group (N = 3354)	0.02 (0.02-0.02)	0.33 (0.30-0.37)	0.03 (0.03-0.03)	0.24 (0.23-0.25)
	≤ 1 month (N = 454)	0.02 (0.02-0.03)	0.20 (0.17-0.21)	0.03 (0.03-0.04)	0.16 (0.14-0.18)
	> 1 month - ≤ 1 year (N = 1030)	0.02 (0.02-0.03)	0.28 (0.25-0.33)	0.03 (0.03-0.03)	0.21 (0.20-0.24)
	1-5 years (N = 1015)	0.02 (0.02-0.02)	0.33 (0.30-0.40)	0.03 (0.03-0.03)	0.23 (0.22-0.26)
	5-18 years (N = 855)	0.02 (0.02-0.03)	0.8 (0.34-0.41)	0.03 (0.03-0.04)	0.28 (0.26-0.30)
C10:1	All group (N = 3356)	0.02 (0.02-0.03)	0.28 (0.27-0.28)	0.04 (0.04-0.04)	0.22 (0.22-0.23)
	≤ 1 month (N = 454)	0.02 (0.02-0.03)	0.23 (0.21-0.24)	0.04 (0.03-0.04)	0.17 (0.15-0.190)
	1-5 years (N = 2046)	0.03 (0.02-0.03)	0.26 (0.25-0.29)	0.04 (0.03-0.04)	0.22 (0.21-0.23)
	5-18 years (N = 856)	0.02 (0.02-0.03)	0.29 (0.28-0.32)	0.04 (0.04-0.05)	0.25 (0.24-0.27)
C12	All group (N = 3322)	0.02 (0.01-0.02)	0.16 (0.16-0.17)	0.03 (0.03-0.03)	0.13 (0.13-0.13)
	≤ 1 month (N = 438)	0.02 (0.02-0.03)	0.18 (0.17-0.19)	0.03 (0.03-0.04)	0.15 (0.14-0.16)
	> 1 month - ≤ 1 year (N = 1024)	0.02 (0.02-0.02)	0.16 (0.16-0.18)	0.03 (0.03-0.03)	0.13 (0.13-0.14)
	1-18 years (N = 1860)	0.01 (0.01-0.02)	0.16 (0.14-0.17)	0.02 (0.02-0.03)	0.12 (0.12-0.13)
C14	All group (N = 3354)	0.03 (0.03-0.04)	0.33 (0.32-0.37)	0.05 (0.04-0.05)	0.25 (0.24-0.26)
	≤ 1 month (N = 454)	0.03 (0.03-0.04)	0.42 (0.40-0.47)	0.06 (0.05-0.06)	0.33 (0.32-0.38)
	> 1 month - ≤ 1 year (N = 1029)	0.04 (0.03-0.05)	0.32 (0.31-0.36)	0.05 (0.05-0.06)	0.27 (0.25-0.28)
	1-5 years (N = 1016)	0.03 (0.03-0.04)	0.23 (0.21-0.26)	0.04 (0.04-0.05)	0.19 (0.18-0.20)
	5-18 years (N = 855)	0.03 (0.02-0.03)	0.12 (0.18-0.24)	0.04 (0.04-0.04)	0.16 (0.15-0.17)
C14:1	Al group (N = 3305)	0.02 (0.02-0.02)	0.17 (0.16-0.18)	0.02 (0.02-0.02)	0.14 (0.14-0.15)
	≤ 1 month (N = 443)	0.01 (0.01-0.02)	0.19 (0.18-0.23)	0.03 (0.02-0.03)	0.15 (0.14-0.15)
	>1 month - ≤ 1 year (N = 1017)	0.02 (0.02-0.02)	0.16 (0.16-0.18)	0.03 (0.02-0.03)	0.14 (0.14-0.15)
	1-5 years (N = 1001)	0.01 (0.01-0.02)	0.17 (0.16-0.19)	0.02 (0.02-0.02)	0.14 (0.14-0.15)
	5-10 years (N = 511)	0.00 (0.00-0.00)	0.15 (0.14-0.16)	0.02 (0.02-0.03)	0.15 (0.14-0.16)
	10-18 years (N = 333)	0.01 (0.01-0.02)	0.25 (0.19-0.27)	0.02 (0.02-0.03)	0.14 (0.13-0.16)
C16*	All group (N = 3349)	0.32 (0.29-0.34)	4.03 (3.67-4.43)	0.43 (0.41-0.44)	2.69 (2.53-2.92)
	≤ 1 month (N = 452)	0.20 (0.18-0.22)	6.70 (6.02-7.39)	0.41 (0.36-0.45)	4.52 (3.79-4.49)
	>1 month - 10 years (N = 2562)	0.33 (0.29-0.35)	2.76 (2.43-3.19)	0.43 (0.41-0.44)	1.90 (1.84-1.99)
	10-18 years boy (N = 196)	0.42 (0.38-0.46)	2.20 (2.02-2.40)	0.50 (0.47-0.54)	1.83 (1.61-1.95)
	10-18 years girl (N = 139)	0.30 (0.24-0.38)	2.51 (2.03-3.14)	0.34 (0.10-0.47)	1.55 (1.31-2.14)
C16:1	All group (N = 3266)	0.00 (0.00-0.00)	0.23 (0.20-0.30)	0.01 (0.01-0.02)	0.14 (0.13-0.14)
	≤ 1 month (N = 393)	0.00 (0.00-0.00)	0.21 (0.19-0.23)	0.01 (0.01-0.03)	0.25 (0.20-0.32)
	>1month - ≤ 1 year (N = 1011)	0.00 (0.00-0.00)	0.17 (0.15-0.53)	0.01 (0.01-0.02)	0.14 (0.13-0.14)
	1-18 years (N = 1862)	0.00 (0.00-0.00)	0.15 (0.14-0.15)	0.01 (0.01-0.02)	0.12 (0.12-0.13)
C16-OH	All group (N = 2271)	0.01 (0.00-0.01)	0.07 (0.06-0.08)	0.01 (0.01-0.01)	0.05 (0.04-0.05)
	≤ 1 month (N = 325)	0.00 (0.00-0.00)	0.05 (0.05-0.06)	0.01 (0.01-0.01)	0.05 (0.05-0.06)
	> 1month - 5 years (N = 689)	0.01 (0.00-0.01)	0.07 (0.05-0.08)	0.01 (0.01-0.01)	0.04 (0.04-0.05)
	1-18 years (N = 1257)	0.01 (0.00-0.01)	0.07 (0.06-0.10)	0.01 (0.01-0.01)	0.04 (0.04-0.05)
C18	All group (N = 3354)	0.17 (0.15-0.18)	1.58 (1.50-1.64)	0.24 (0.23-0.24)	1.21 (1.18-1.24)
	≤ 1 month (N = 454)	0.15 (0.13-0.16)	2.02 (1.83-2.17)	0.20 (0.18-0.23)	1.41 (1.27-1.54)
	> 1 month - ≤ 1 year (N = 1030)	0.15 (0.11-0.17)	1.56 (1.26-1.65)	0.21 (0.20-0.22)	1.15 (1.10-1.21)
	1-5 years (N = 1015)	0.22 (0.16-0.24)	1.58 (1.42-1.70)	0.30 (0.27-0.32)	1.22 (1.18-1.33)
	5-18 years (N = 855)	0.19 (0.14-0.23)	1.56 (1.37-1.80)	0.28 (0.25-0.31)	1.18 (1.13-1.24)
C18:1	All group (N = 3277)	0.23 (0.19-0.27)	2.76 (2.66-2.84)	0.44 (0.42-0.46)	2.24 (2.17-2.32)
	≤ 1 month (N = 445)	0.27 (0.24-0.29)	3.78 (3.42-4.09)	0.36 (0.32-0.40)	2.58 (2.36-2.67)
	> 1-≤ 1 year (N = 2832)	0.24 (0.19-0.32)	2.75 (2.57-2.87)	0.46 (0.44-0.48)	2.17 (2.11-2.27)
C18-OH	All group (N = 2214)	0.00 (0.00-0.00)	0.04 (0.03-0.04)	0.00 (0.00-0.00)	0.03 (0.02-0.03)
	≤ 1 month (N = 321)	0.00	0.04	0.00 (0.00-0.01)	0.03 (0.02-0.04)
	>1 month - ≤ 1 year (N = 669)	0.00 (0.00-0.00)	0.02 (0.02-0.03)	0.00 (0.00-0.01)	0.02 (0.02-0.03)
	1-18 years (N = 1224)	0.00 (0.00-0.00)	0.04 (0.03-0.04)	0.00 (0.00-0.00)	0.03 (0.02-0.03)
*Difference across gender groups. CI - confidence interval.					

## Discussion

Establishing age- and gender-specific RIs that account for changes related to growth and development is essential for paediatric population. This study is the first to evaluate age and gender-specific RIs for 10 amino acids and 22 acylcarnitines using MS/MS in Turkish paediatric population.

In this study, the majority of metabolites did not show significant gender differences. It is claimed that gender-dependent variations are very rare and most studies have not established gender-specific RIs (*6*). However, a study conducted in Colombia on 891 healthy newborns aged 1-18 days reported that boys had higher concentrations of arginine in the amino acid group and higher concentrations of C0 and C14 in the acylcarnitines group (*1*). Cavedon *et al.* also detected minor gender-dependent variations in C14, C16 and C18:1 concentrations, but detailed results about differences were not reported in the manuscript (*17*). Another study investigating age- and gender-specific variations showed that serum C0 concentrations were related to age and sex, but serum acylcarnitine concentrations remained constant (*18*). Between ages of 15 and 50, males were found to have considerably higher C0 concentrations than females, and a significant negative correlation between serum C0 concentrations and female estradiol concentrations was discovered (*18*). The authors hypothesized that estrogen lowers blood C0 concentrations and contributes to sex-related variations. Due to metabolic, hormonal, and nutritional changes that occur from fetal development to maturity, certain metabolites may show gender variations in addition to age-related differences, and these variations should be researched across a range of age groups.

In a research investigating age-related variations in acylcarnitine concentrations, similar to ours, C0 was shown to be considerably higher in older children than in newborns, while the concentrations of various other acylcarnitines tended to be significantly lower in cord blood and older children’s groups than in the control group (3-6 days) (*17*). The lower cut-offs for acylcarnitines in advanced age may pose challenges in diagnosing carnitine deficiency. Age- and gender-specific cut-offs for acylcarnitines could be crucial, especially when the diagnostic cut-off for a metabolic disorder is very close to the reference interval. For instance, in individuals with long-chain acylcarnitine dehydrogenase deficiency, even slight increases in long-chain acylcarnitine concentrations may be indicative of the disease (*17*, *19*).

In our study, C16 concentrations were found to be considerably lower in the 11-18 years age group than in the ≤ 1 month infants. If those older children were managed, using RIs established for the ≤ 1 month age group, there might have been a potential for missed diagnosis or undertreatment. Age-related RIs are especially crucial for examination of siblings who have a stable clinical state and a medium-chain acylcarnitine deficiency since these patients do not show a marked increase in the acylcarnitine profile unless they experience metabolic stress (*20*). Additionally, if age-related RIs are not employed, characterizing older individuals might be difficult since the increase in acylcarnitine concentration may be modest in patients who are clinically compensated or in a fed state (*20*). While the concentration of acylcarnitines is markedly elevated in some metabolic disorders, such as propionic aciduria, isovaleric aciduria, and carnitine palmitoyl transferase deficiency, the small but statistically significant differences related to age might not have a significant impact on the clinical diagnosis (*19*).

In a study by Dogan *et al.* from Turkey, the MS/MS results of 4800 children under or over one year of age were downloaded from LIS, and the RIs were established using the indirect method. Although the authors observed age-dependent differences in RIs, they concluded that this did not influence diagnosis of the most common IEM because the majority of patients exhibited noticeable increases in either amino acid or acylcarnitine concentrations (*14*). Demirelce *et al.* also evaluated the MS/MS results of 9994 newborns retrospectively and presented the 1st and 99th percentile distribution as well as the mean + 4SD results of their data. Additionally, they reported incidence rates, positive predictive values, and false-positive rates for 26 screened disorders. Their 99th percentile results were similar to our newborn results for most of the amino acids and acylcarnitines but lower than those reported by the CDC. They concluded that development of a nationwide screening panel is necessary for early detection and management of IEM (*15*). Macit *et al.* established age- and sex-specific reference intervals for plasma and urine amino acids in a total of 945 clinically healthy Turkish paediatric populations ranging in age from birth to 14 years using high-performance liquid chromatography. They reported significant sex-related differences, with higher concentrations of essential amino acids in plasma in girls than in boys in the age groups of 0-1 month and 7-14 years. Concentrations of essential amino acids in urine were higher in girls than in boys in the age group of 0-1 month. However, there was no difference in the other age groups. Their study differed from ours in terms of both method and sample type selection, which might explain differences between results (*21*).

In studies performed on different populations, varying RIs and cut-offs were reported (*1*, *13*, *17*, *22*). One of these studies involved 25-30 million healthy newborns from around the world as part of a global collaborative effort (*23*). However, the cut-offs for amino acids and acylcarnitines depend on several factors, such as age, medication usage, parenteral nutrition, testing principle, instrument used, and genetic or ethnic background of a particular population (*5*, *22*, *23*). According to CLSI, each laboratory should establish its own RIs and cut-offs following guidelines approved by CLSI. One of the main challenges in RI investigations is the collection of samples from healthy individuals. On the other hand, the indirect method, which involves a large data set and considers population heterogeneity, is a valid and recommended approach for determining population-based RIs (*8*, *9*).

This study is significant for establishing both age- and gender-specific RIs and cut-offs for amino acids and acylcarnitines in Turkish population. Reference intervals should be established as part of a national initiative involving a larger number of individuals and centers. One of the limitations of our study was that analysis were not done in duplicate. It was conducted in a single center, and the sample size was relatively small for some age and gender subgroups. Another limitation is the absence of ratios, which have been shown to improve diagnostic sensitivity or specificity and aid in treatment monitoring. Additionally, we were unable to assess clinical utility of RIs we established.

In conclusion, establishing age-related RIs can enhance the quality of medical care by facilitating early diagnosis and therapy, especially in certain metabolic disorders presenting with mild biochemical abnormalities and subtle clinical manifestations.
